# Management of Crush Syndrome Casualties after Disasters

**DOI:** 10.5041/RMMJ.10039

**Published:** 2011-04-30

**Authors:** Mehmet Sukru Sever, Raymond Vanholder

**Affiliations:** 1 Local co-ordinator for the Renal Disaster Relief Task Force of the International Society of Nephrology (ISN); Department of Internal Medicine/Nephrology, Istanbul School of Medicine, Istanbul, Turkey, and; 2 Chairman, Renal Disaster Relief Task Force of the ISN; Renal Division, Department of Internal Medicine, University Hospital, Ghent, Belgium

**Keywords:** Crush syndrome, rhabdomyolysis, kidney, trauma

## Abstract

After direct impact of the trauma, crush syndrome is the second most frequent cause of death after mass disasters. However, since crush syndrome is quite rare in daily practice, mistakes are frequent in the treatment of these cases. This paper summarizes the etiopathogenesis of traumatic rhabdomyolysis and of crush syndrome-based acute kidney injury. The clinical and laboratory features, prophylaxis, and treatment of crush cases are described as well. The importance of early and energetic fluid resuscitation is underlined for prophylaxis of acute kidney injury. Since there is chaos, and an overwhelming number of victims, logistic drawbacks create a specific problem in the treatment of crush victims after mass disasters. Potential solutions for logistic hurdles and disaster preparedness scenarios have also been provided in this review article.

## INTRODUCTION

Literally, the word “crush” means “compression between opposing elements so as to break or injure”. In medicine, an association between compression trauma and renal failure was first described after the Messina earthquake in Sicily in 1909 and after World War I in the German military literature.^1^ However, the full scope of renal failure after crush was first recognized as an entity by Bywaters and Beall in 1941 in the victims of the blitz of London.^2^ Although crush injury refers only to trauma, the term *crush syndrome* indicates systemic manifestations of muscle crush injury after direct trauma or ischemia reperfusion injury.^3^ Such manifestations may include tense, edematous, and painful muscles, hypovolemic shock, acute kidney injury (AKI), hyperkalemia, acidosis, cardiac failure, respiratory failure, and infections.^4^

The primary event in crush syndrome is rhabdomyolysis, which is “disintegration of striated muscles that results in release of muscular cell contents into the extracellular fluid”.^5^ Among these substances, lactic acid, thromboplastin, creatine kinase, nucleic acids, phosphate, and creatine can be cited, while the most important ones are myoglobin and potassium. In addition to hypovolemia, these substances play an important role in the pathogenesis of crush syndrome.

Striated muscles are located in the spaces or “compartments” formed by rigid, non-compliant fascias. Normally, the pressure in these spaces is very low (i.e. 0–20 mmHg). An increased pressure in the compartment that disrupts the perfusion and hinders the function of the tissues is referred to as “compartment syndrome”. In other words, compartment syndrome is a muscle tamponade. If intracompartmental pressure is left to increase without any treatment, tissue necrosis can develop, which may result in deterioration in the clinical and laboratory findings of the patient.

## DISASTERS AND CRUSH SYNDROME

Disasters cause material as well as structural losses, and damage to the infrastructure. It is fairly impossible to prevent or even anticipate these disasters, although major morbidity and mortality are often the unfortunate consequence. Following these catastrophes, especially earthquakes, collapsing structures of buildings can hit vital organs such as the brain, lungs, and liver, hence causing instant death. Alternatively, these materials can also compress non-vital organs such as the muscles, resulting, as a consequence, in rhabdomyolysis, the crush syndrome, and AKI as less immediate complications being diagnosed hours to days after the initial event.^5^

It has been reported that in the case of a sudden collapse of an eight-story building, 80% of the entrapped victims instantly die by the direct effects of trauma, 10% survive with minor trauma, while 10% are badly injured; of those, 7/10 develop crush syndrome.^6^ If these observations are extrapolated to earthquakes whereby numerous buildings collapse, dramatic numbers of crush victims can be expected. Apart from direct impacting trauma to the chest, head, or abdomen, the crush syndrome is the most frequent cause of mortality in survivors of earthquakes.^7^

Since it is almost impossible to prevent instant deaths, virtually the only way to decrease mortality during extensive catastrophes is to manage the seriously injured victims with sub-acute problems properly. Among those, patients with the crush syndrome and/or AKI constitute an important group, especially since proper dialysis treatment until renal function recovers is one of the most relevant approaches, if not the only one, to save the lives of patients that would otherwise die.

Earthquakes of high magnitude with large numbers of victims have always occurred during the entire history. Because of the lack of extended dialysis facilities, however, the therapeutic aspects of massive crush with subsequent kidney failure in large numbers of subjects had never been reported until recently. The first catastrophe of epidemic dimensions ever described occurred in the aftermath of the Armenian earthquake in the late 1980s.^8^ Since then, at least 10 mass disasters were registered with the potential to cause substantial numbers of crush victims and/or subjects needing dialysis, and, in most of these instances, the presence of crush and/or renal failure was acknowledged (Table 1).^9^–^11^

It is well known that many earthquake-prone areas lie in densely inhabited regions, amongst which the Californian fault and the whole Mediterranean area. Both Istanbul and Tehran, two cities with more than 10,000,000 inhabitants, are situated very close to a fault. Preventive conceptual thinking regarding this matter is urgently needed since the risk for a major earthquake in those cities and areas is extremely high, e.g. for Istanbul 32% ± 12% until 2011 and 62% ± 15% until 2031.^12^

## THE CONCEPT OF “RENAL DISASTER”

Calculated/registered numbers of crush syndrome victims after earthquakes have been reported to be as high as 3,000, 600, and 639 after the Tangshan-China, Armenian, and Marmara–Turkey earthquakes, respectively;^8^,^13^,^14^ hence, in addition to the primary catastrophe, a subsequent one may be added which has been named “renal disaster”.^15^ Since treatment of crush-related AKI is highly complicated, most of the time medical and logistic problems cannot be coped with locally, and material and personnel support is needed. However, poorly organized relief worsens the chaos, and creates a “second disaster”, interfering with other global rescue activities.^15^ The disappointing experiences after the Armenian earthquake stimulated the International Society of Nephrology (ISN) to install the “Renal Disaster Relief Task Force” (RDRTF) as a logistic organization to avoid similar problems in future disasters.^16^ The Marmara earthquake, in Turkey, was the first large-scale catastrophe to put the organizational structure of the ISN-RDRTF mentioned above into action and to test its effectiveness, because in this catastrophe the number of totally collapsed or heavily damaged buildings exceeded 100,000, and in total 639 victims with acute renal problems related to crush syndrome were registered (Table 1).^14^ Both useful contributions to local disaster organization as well as effective material, personnel, and moral help, which had been planned far in advance, very likely have contributed to the strikingly lower mortality rates noted in this catastrophe as compared to mortality rates in the crush victims of the Japan Kobe earthquake.^17^

## ETIOLOGY IN RHABDOMYOLYSIS-RELATED AKI

Rhabdomyolysis may result from both non-traumatic and traumatic etiologies (Table 2). Although non-traumatic causes are more common in daily life, traumatic etiology becomes more prominent following extraordinary events such as mine collapses, traffic accidents, wars, and natural and man-made (artificial) disasters.

Pathogenesis of crush syndrome can be studied under two headings:^18^,^19^ pathogenesis of traumatic rhabdomyolysis and pathogenesis of rhabdomyolysis-induced AKI.

## PATHOGENESIS OF TRAUMATIC RHABDOMYOLYSIS

This involves one or a combination of the following mechanisms: 1) inadequate supply of adenosine triphosphate (ATP), 2) sustained increments in sarcoplasmic calcium concentration, and 3) increased permeability of the sarcolemma.

**Figure 1:**
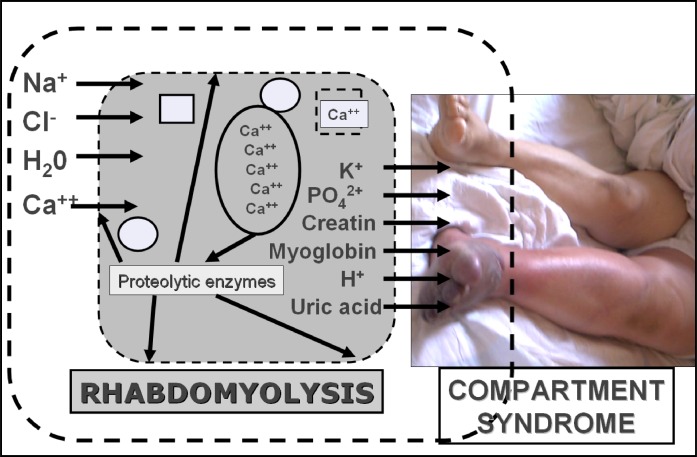
Pathogenesis of pressure-induced rhabdomyolysis (based on reference 1).

Other contributing mechanisms include: ischemia reperfusion pathway and inflammatory processes, which can generate reactive oxygen species, and diverse cytokines. Mostly, these mechanisms act in combination and are similar among various etiologies. Below, as a prototype, pathogenesis of pressure-induced rhabdomyolysis will be described (Figure 1):^20^

When muscles are compressed, the permeability of the sarcolemma increases, and substances abundant in the extracellular environment such as calcium, sodium, and water move to the intracellular milieu, while substances high in the muscle cells (such as potassium and myoglobin) efflux to the extracellular environment. Once a critical free calcium concentration is reached, sustained muscle contraction ensues and depletes ATP stores; mitochondrial damage occurs resulting in oxidant stress; and proteases, phospholipases, and other enzymes are activated, resulting in myofibril and membrane phospholipid damage.^18^ The net result is myocyte lysis and release of toxic intracellular constituents into the extracellular microenvironment.

Local accumulation of these products causes microvasculature damage, producing capillary leak, subsequently causing compartmental syndrome, which increases pressure on the capillaries triggering occlusion of the microcirculation and rapidly depleting myoglobin oxygen content. Similarly, creatine, phosphate, and glycogen stores are exhausted as well, and severe ATP depletion ensues.^18^ However, in ischemic tissue injury most of the damage occurs after flow into the damaged tissue is restored. In this case, leukocytes migrate into these particular tissues after reperfusion has started, and production of free radicals starts after oxygen is available (reperfusion injury).

## PATHOGENESIS OF RHABDOMYOLYSIS-INDUCED AKI^18^,^20^,^21^

**Figure 2:**
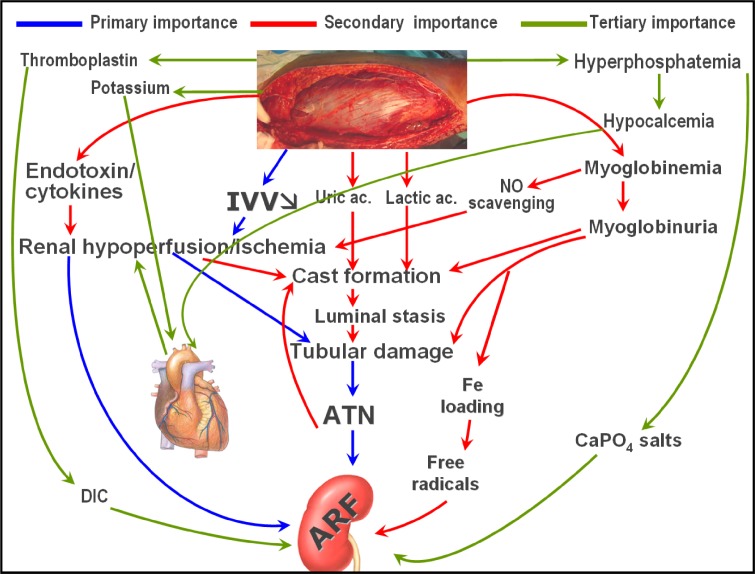
Pathogenesis of crush syndrome-related acute kidney injury (adapted from references 19–21). ARF, acute renal failure; ATN, acute tubular necrosis; DIC, disseminated intravascular coagulation; IVV, intravascular volume.

Several mechanisms contribute to AKI in this setting (Figure 2): 1) Muscle necrosis causes dramatic fluid third spacing, leading to intravascular volume depletion, renal hypoperfusion, and ischemia. AKI is prerenal at the beginning; how-ever, if not treated properly, acute tubular necrosis (ATN) can develop. 2) Myoglobin is released from traumatized muscles, and subsequent myoglobinuria causes intratubular cast formation which contributes to AKI. 3) Myoglobin scavenges nitric oxide (NO), that aggravates renal hypo-perfusion and tissue injury. 4) Severe muscle injury can activate the endotoxin-cytokine cascade; subsequent renal vasoconstriction contributes to renal hypoperfusion and ischemia. 5) Nucleosides released from disintegrating cell nuclei are metabolized in the liver to uric acid, which may contribute to cast formation and tubular obstruction. Degradation of intratubular myoglobin causes release of free iron, which catalyzes free radical production, enhancing ischemic damage. 7) Potassium released from the damaged muscles depresses cardiac output, potentiating renal hypoperfusion. 8) Hyperphosphatemia may contribute to hypocalcemia, which can further depress myocardial contractility. 9) Hyperphosphatemia may result in the precipitation of CaPO_4_ salts that induce inflammation of the kidney tissue. 10) Damaged muscles can release tissue thromboplastin, triggering disseminated intravascular coagulation that contributes to AKI.

## CLINICAL AND LABORATORY FINDINGS

The *clinical spectrum* of rhabdomyolysis varies from asymptomatic elevation in creatine kinase to acute oliguric ATN and multi-organ failure.

Overall, clinical findings can be classified as: 1) local findings in the traumatized muscles, which include pain, pressure, paresthesia, paresis or paralysis, pallor, and pulselessness (the six “P”s), and 2) systemic findings (or findings of crush syndrome). Crush syndrome develops in 30%–50% of rhabdomyolysis cases, and symptoms include hypovolemic shock, hyperkalemia, heart failure, respiratory failure, infections, and, importantly, AKI.^10^

*Laboratory findings* of rhabdomyolysis can be discussed under two headings of urinary findings and biochemical features.

Typical finding in urinalysis is a dirty-brownish discoloration of the urine as a result of myoglobinuria. Macroscopic hematuria and trace proteinuria may also be observed.

Biochemical features are related to increased serum levels of substances released from the injured muscles, such as increased urea, creatinine, phosphate, potassium, and muscle enzymes and acidosis. Among these, hyperkalemia is the most critical parameter and results in many patient deaths.^22^

## PROPHYLAXIS AND TREATMENT

Rhabdomyolysis is necrosis of the muscles; hence no specific treatment can reverse this process. However, if applied early and energetically, some interventions may limit the progression of the pathology and prevent complications such as crush syndrome. Below, treatment of entrapped crush casualties will be described.

It has been suggested that when an extremity of a living victim is detected under the rubble, if possible, an infusion of isotonic saline at a rate of 1 L/hour should be initiated.^19^ After extrication, hydration status of the victim should be evaluated to determine the volume of fluids required. If no intravenous fluid had been given prior to rescue, isotonic saline at a rate of 1,000 mL/h for adults (15–20 mL/kg/h for children) should be initiated. Once the victim has received 6 L of fluid, either before or after extrication, urine output and volume status should be evaluated to determine the further amount of fluid to be given.

For defining further fluid administration protocol, in addition to amount of urine response to fluid resuscitation, the following issues should be considered.

### Purpose

The first priority is volume resuscitation and repletion, which is critical to reverse hypovolemic shock, prevent AKI, and thereby minimize lactic acidosis and hyperkalemia. The second priority is systemic alkalinization as a means to reduce acidosis and hyperkalemia. Reducing intracompartmental pressures by medical means is also important.

### Choice of fluids

*Isotonic saline* is effective for volume replacement and prevention of AKI; it is the most likely readily available solution and carries the lowest risk of side-effects in the chaos of mass disasters.

If available, *5% dextrose + isotonic saline solution* should be administered, which may provide the advantage of supplying some calories and attenuating hyperkalemia.

*Sodium bicarbonate, added to half-isotonic solutions* may be effective for alkalinizing urine above 6.5 to prevent renal tubular deposition of myoglobin and uric acid, to improve metabolic acidosis and reduce hyperkalemia.^19^ Alkaline solutions should be administered to all victims in small-scale disasters, unless symptomatic alkalosis, suggested by the presence of neuromuscular irritability, somnolence, or paresis, is present. Excessive alkalinization has drawbacks, however, such as the promotion of symptomatic alkalosis, calcium phosphate deposition in soft tissues, worsening of hypocalcemia, and volume overload.

*Mannitol* has diuretic, antioxidant and vasodilatory effects and, because of its tonicity, decreases muscle intracompartmental pressure.^23^ Mannitol may also be useful in crush casualties by expanding extracellular volume, increasing urine output, and preventing renal tubular cast formation. However, considering side-effects (congestive heart failure in the case of overdose, and potential nephrotoxicity)^24^ as well as inconsistent reports of efficacy in traumatic rhabdomyolysis,^25^ there is no consensus regarding mannitol administration. Mannitol is discouraged in anuric patients.

*Colloids* can be used as initial management for expansion of intravascular volume in patients at risk of or with AKI. On the other hand, crystalloids are generally preferred over colloids for fluid resuscitation considering no major benefit of colloids on morbidity and mortality, a higher risk of side-effects such as anaphylaxis or coagulation abnormalities, a risk of AKI at high doses (starch preparations), and higher costs.^26^

### Application

Addition of bicarbonate to hypotonic solutions makes them almost isotonic. The average need for bicarbonate is 200–300 mEq/day.

If mannitol is to be used, 60 mL of 20% mannitol (overall 12 g, or 200 mg/kg) is given intravenously over 3–5 min as a test dose to observe urine response.^23^ If there is no significant increase in the urine output, mannitol should not be continued. However, if urine output increases by at least 30–50 mL/h above base-line levels, mannitol may be added to the solutions mentioned above. The usual dosage of mannitol is 1–2 g/kg per day (total, 120 g/day) at a rate of 5 g/h.^10^

Mannitol-alkaline solution can be applied up to 12 L/day to an adult. In the crush victims of Bingol–Turkey^27^ and Kobe–Japan^28^ earthquakes volumes of administered fluids reached even more than 20 L/day with very favorable results. However, in chaotic circumstances of mass disasters, these quantities may carry a risk of volume overload. The best approach would be to individualize fluid policy and consider both medical and logistic factors when planning fluid resuscitation. In general elderly patients should be dosed less aggressively (4–6 L/day) to prevent volume overload.^5^ In victims with compartment syndrome urine response can be significantly lower than administered fluids due to third spacing.

In the case of established anuria after excluding hypovolemia, and no urine response to fluid resuscitation, all fluids should be restricted to 0.5–1 L/day in addition to a volume equivalent to all measured or estimated fluid losses of the previous day.

If fluid resuscitation cannot be performed in the early period, intrarenal AKI, almost always due to acute tubular necrosis (ATN), develops. ATN can be non-oliguric; however, mostly it is characterized by an initial oliguric period that is followed by polyuria. Treatment in the *oliguric period* includes conservative treatment and dialysis. Interventions in the conservative approach include avoiding nephrotoxic insults, maintaining fluid–electrolyte, acid–base balance and prescribing appropriate diet (low protein/potassium and adequate calories). Dialytic interventions include intermittent hemodialysis, slow continuous therapy, and peritoneal dialysis. Among these, hemodialysis is preferred because of high clearance and logistic advantages.^5^

Dialysis indications do not differ from daily practice: crush victims should be dialyzed in the presence of clinical symptoms such as hypertension, volume overload, nausea, and/or biochemical abnormalities such as severe uremia, hyperkalemia, acidemia. Also, “prophylactic dialysis” should be performed in patients with high risk for hyperkalemia.

In the Marmara earthquake experience 477 of the patients needed renal replacement therapy (RRT). Intermittent hemodialysis (IHD) was the most commonly applied treatment; of the patients who received IHD most needed 1–15 sessions for a 1–15-day period. In total 5,137 sessions of hemodialysis were performed, which underlines this intervention as the largest acute hemodialysis intervention reported so far.^29^

Treatment in the *polyuric period* includes prescribing appropriate diet and maintaining acid–base and fluid–electrolyte balance. If appropriate amounts of fluids are not given, renal perfusion may become impaired again, and prerenal or even intrarenal AKI may re-emerge.

An unresolved issue in traumatic rhabdomyolysis cases is fasciotomies. It may have beneficial effects, because decompression may restore circulation and decrease necrotic muscle mass, thus preventing AKI and irreversible neurological damage.^30^,^31^ However, it has drawbacks as well, such as turning a closed injury into an open wound that results in infection risk and severe disabilities in the long term.^4^,^9^,^32^ In the Marmara earthquake crush syndrome victims, overall 397 fasciotomies were performed in 323 patients; 25% of the fasciotomized patients were complicated by sepsis, while only 13% of the non-fasciotomized victims suffered from this complication. The mortality rate of the patients with sepsis was higher as compared to the non-septic victims. Therefore, although it can be very beneficial, fasciotomy is a risky intervention in crush syndrome casualties of massive earthquakes, hence should be performed only by objective criteria such as intracompartmental pressure measurements.^5^

## LOGISTIC PROBLEMS IN TREATING CRUSH SYNDROME VICTIMS

Literally, the word logistics means “the procurement, maintenance, distribution, and replacement of personnel and material”. Although usually not considered in the routine daily practice, logistic planning after catastrophic earthquakes is vital for providing the most effective treatment, because this time-period is characterized by chaos and shortage of medical material and personnel.

In order to reduce the chaos, logistic planning can be described on two main levels: the global (or the international) level and the local (or the national) level.

## GLOBAL LOGISTIC PLANNING^10^

In the case of an earthquake, the Chairman of the RDRTF is informed by US geological services. Afterwards:

- He/she estimates dimensions of the disaster and defines need for an international relief intervention
- A scouting team is sent to the disaster region
- Primary information is relayed back to the RDRTF Chairman, to mobilize additional teams and supplies
- A key person from the affected country is identified
- This key person, in conjunction with the Chairman of RDRTF, will be responsible for the local co-ordination
- The local co-ordinator firstly reports local conditions to the Chairman of the Task Force, then estimates dimensions of the problem and anticipates the need for support
- Informs Chairman of the Task Force for international support and local authorities for national support
- Then, support is offered if needed.

## LOCAL LOGISTIC PLANNING

*Local logistic actions* after renal disasters can be described under the headings of: I. Severity assessment; II. Providing health care to the casualties; and III. Medical support.^10^

### 

#### I. SEVERITY ASSESSMENT

This is vital to estimate the need for national and international support. Following massive earthquakes, the ratio of deaths to the injured is one death for every three injured casualties,^33^ and, overall, 2%–3% of all casualties can be expected to be complicated by crush syndrome. This rate seems quite low, but in the case of thousands of wounded, it is apparent that crush syndrome is a major cause of deaths. On the other hand, these estimations may not be valid for all disasters, because numerous factors take part, such as intensity of the disaster, population density of the region, structural characteristics of buildings, and timing (or even the moment) of disaster. These variables deeply influence the number of casualties. For example, in the Gujarat earthquake in India, in 2001, the death toll was around 20,000, but the number of crush cases was only 35. This low number was explained by the day-time occurrence of the disaster, thus the majority of deaths occured instantly due to head traumas.^10^ In the Bam earthquake in Iran, the death toll was around 26,000, but crush syndrome cases numbered only 124; very probably earth-made buildings caused instant suffocation and death of the casualties. Another similar example is the unexpectedly low number of crush casualties following the September 11 terrorist attack in US. After this violence, the total number of deaths was more than 3,000, while AKI due to crush syndrome was diagnosed only in 1 case. This finding was explained by the severity of the disaster resulting in so many instant deaths due to the fire and sudden collapse and a very few injured victims.^34^

#### II. PROVIDING HEALTH CARE TO THE CASUALTIES

*This can be summarized under the headings of* rescue activities, transport of the victims, and logistic planning in hospitals.

*Rescue activities* are of major importance following earthquakes. Considering the rescuers, it is well known that the most effective rescue work after earthquakes is not accomplished by trained teams but by ordinary people or other surviving casualties. According to a retrospective analysis conducted after the Armenian earthquake, the majority of the survivors were rescued by their untrained neighbors who had survived the earthquake with no major traumas. In this analysis, it was found that only 2.6% of the casualties were extricated by the Russian experts and less than 1% was rescued by foreign teams.^35^ On the other hand, in the Southern Italian earthquake, only 18% of the uninjured people (neighbors) took part in the rescue activities; this lack of concern was attributed to the probable psychological shock following the disaster and the lack of education.^36^ Therefore, the media in disaster-prone regions should make programs that draw the attention of the public to this vital issue and encourage citizens to take part in rescue activities. People living in disaster-prone regions should consider that they are needed as “rescuers” in the case of a disaster.

*Transport of the victims* away from the disaster area is essential after mass disasters for several reasons: 1) frequent aftershocks may damage hospitals and dialysis centers, 2) it is necessary to keep positions open in the local hospitals for cases who cannot be transported, and 3) locally treated patients have a higher risk of mortality compared to victims treated in cities distant to the disaster area.^37^

On the other hand, transport of victims in disaster conditions may be problematic. Therefore, alternative means for transporting the patients should be used such as helicopter, or boats, if applicable.

*Logistic planning in hospitals* is critical in treatment of disaster victims, because thousands of complicated cases may need medical care, while there may be considerable damage to hospital stocks. Therefore, until effective help is received, careful consumption of available stock is important, which is possible only by anticipating the timing of admissions.

Most hospital admissions occur within the first three days of the disaster.^14^ Hospital beds should be used very carefully as well; mildly injured victims deserve special mention because they can arrive shortly after disaster by their own means, occupying positions of more seriously wounded cases, who often arrive later. Therefore, health care personnel should consider that not all victims arriving at the emergency room need to be hospitalized; rather, mildly injured victims should be referred to their homes to be followed on an out-patient basis.

Another problem in providing health care to disaster victims is inefficiency of the health care personnel because of personal harm to themselves or family members, work overload, and the panic and depression that they are faced with.^7^,^38^

In order to ensure utmost efficiency and to minimize the risk of malpractice in disaster conditions, experienced personnel should be assigned on duty within the first days, when more complicated cases are expected; non-stop work during the first days should be avoided to prevent “burnout” syndrome; and clear guidelines should be prepared.^39^

Last, but not least, to reduce the dimensions of chaos in the post-disaster period, in earthquake-prone areas, macroplanning of the medical personnel should be prepared in advance, and physicians who will work in collaboration with the rescue teams in the field, in the emergency units of the hospitals, in providing clinical follow-up of the patients, and in logistic co-ordination should be identified and provided special training courses.^39^

#### III. MEDICAL SUPPORT

Since the health care system in the affected regions may not cope with the problems, national and international medical support is often needed. However, international relief is not always functional. In the Guatemalan earthquake in 1976 more than 90% of the medical items were useless because they were unsorted.^40^ In the Armenian earthquake in 1989, 70% of provided drugs were useless because they were expired or damaged.^41^

The same concerns may also be valid for personnel support: this intervention may be useful or useless, or even harmful. Unprepared and inexperienced foreign personnel may hamper relief by tying up communications, transportation, and housing. Therefore, integrated responses of national and international organizations are needed.

When making help calls, it is necessary to estimate the amount of items needed. We defined the approximate amounts of some medical items that would be necessary in the treatment of crush cases considering the experience after the Marmara earthquake.^10^ The mean total volume of crystalloids administered to crush victims during the first day of admission was more than 5,000 mL/patient. Extrapolating this amount to an entire week for a number of 3,000 victims (potential crush victims of the Istanbul earthquake), more than 100,000 L of fluids should be stocked. Also substantial amounts of intestinal potassium binders should be foreseen. At a current dosage of 15 g/day kayexalate for a disaster with 3,000 victims, the amount needed can be calculated to be 315 kg over 1 week. Considering a mean of 11 sessions of dialysis for each crush patient and 75% of the patients needing dialysis support, nearly 25,000 sets of dialysis material would be needed. Same concerns may be applied for blood and blood product transfusions; considering the figures of the Marmara earthquake, for a similar number of crush cases overall 39,000 units blood and blood products would be needed.

As can be noted, it is very hard, even impossible to stock this material before disasters. Therefore, again, effective national and international organized support is of vital importance for saving as many lives as possible.

To conclude, crush syndrome is a major cause of mortality in the rescued victims of massive earthquakes. On the other hand, the number of deaths due to crush syndrome (or fatalities of renal disaster) can be decreased by appropriate management.

Medical practices during disasters differ considerably as compared to routine medical applications. National and international disaster preparedness scenarios and pragmatic logistic planning can be helpful for decreasing the chaos of the post-disaster period, and providing more effective health care services.

## Figures and Tables

**Table 1.** Major earthquakes of the last 20 years with reported statistics in the literature.^9^–^11^

Location, country (year)	Mortality	Crush syndrome	Dialyzed
Spitak, Armenia (1988)	25,000	600	225–385
Northern Iran (1990)	>40,000	(?)	156
Kobe, Japan (1995)	5,000	372	123
Marmara, Turkey (1999)	>17,000	639	477
Chi-Chi, Taiwan (1999)	2,405	52	32
Gujarat, India (2001)	20,023	35	33
Boumerdes, Algeria (2003)	2,266	20 (?)	15 (?)
Bam, Iran (2003)	26,000	124	96
Kashmir, Pakistan (2005)	>80,000	118	65
Sichuan, China (2008)^*^	69,000	?	?
Haiti (2010)	220,000	92	51
*TOTAL*	*> 500,000*	*>2,000*	*>1,200*

* Although many single center reports appeared in the literature, the overall number of crush cases is unknown after this catastrophe.

**Table 2:** Etiology of rhabdomyolysis.^5^

**Non-physical causes**	**Physical causes**
*Electrolyte abnormalities* Hypokalemia, hypocalcemia, hypophosphatemia, hyponatremia, hypernatremia *Alcohol, drugs, and toxins* Regular and illegal drugs Toxins (snake and insect venoms, fish toxins) *Infections and infestations* Infections localized to muscles (pyomyositis) Metastatic infections (sepsis) Other bacterial and viral infections *Metabolic myopathies* Myophosphorylase deficiency (McArdle disease) Other enzymatic defects *Endocrine disorders* Hypothyroidism, diabetic coma *Disseminated intravascular coagulation* *Polymyositis, dermatomyositis*	*Trauma and/or compression of the muscles* Natural and man-made disasters, traffic or working accidents, torture, beating, long-term confinement to the same position *Occlusion or hypoperfusion of the muscular vessels* Thrombosis, embolism, vessel clamping, shock *Electrical current* High-voltage electrical injury Cardioversion *Hyperthermia* High ambient temperatures Neuroleptic malignant syndrome Malignant hyperthermia, sepsis *Strainful exercise* Exercise, delirium tremens, epilepsy

## Abbreviations:

AKI: acute kidney injury;
ATN: acute tubular necrosis;
ATP: adenosine triphosphate;
IHD: intermittent hemodialysis;
ISN: International Society of Nephrology;
NO: nitric oxide;
RDRTF: Renal Disaster Relief Task Force;
RRT: renal replacement therapy.
